# A positive association between nutritional risk and the incidence of surgical site infections: A hospital-based register study

**DOI:** 10.1371/journal.pone.0197344

**Published:** 2018-05-15

**Authors:** Eli Skeie, Anne Mette Koch, Stig Harthug, Unni Fosse, Kari Sygnestveit, Roy Miodini Nilsen, Randi J. Tangvik

**Affiliations:** 1 Department for Research and Development, Haukeland University Hospital, Bergen, Norway; 2 Department of Clinical Science, University of Bergen, Bergen, Norway; 3 Faculty of Health and Social Sciences, Western Norway University of Applied Sciences, Bergen, Norway; 4 Department of Clinical Medicine, University of Bergen, Bergen, Norway; University of Michigan, UNITED STATES

## Abstract

Surgical site infections (SSI) are amongst the most common health care-associated infections and have adverse effects for patient health and for hospital resources. Although surgery guidelines recognize poor nutritional status to be a risk factor for SSI, they do not tell how to identify this condition. The screening tool Nutritional Risk Screening 2002 is commonly used at hospitals to identify patients at nutritional risk. We investigated the association between nutritional risk and the incidence of SSI among 1194 surgical patients at Haukeland University Hospital (Bergen, Norway). This current study combines data from two mandatory hospital-based registers: a) the incidence of SSI within 30 days after surgery, and b) the point-prevalence of patients at nutritional risk. Patients with more than 30 days between surgery and nutritional risk screening were excluded. Associations were assessed using logistic regression, and the adjusted odds ratio included age (continuous), gender (male/female), type of surgery (acute/elective) and score from The American Society of Anesthesiologists Physical Status Classification System. There was a significant higher incidence of SSI among patients at nutritional risk (11.8%), as compared to those who were not (7.0%) (p = 0.047). Moreover, the incidence of SSI was positively associated with the prevalence of nutritional risk in both simple (OR 1.76 (95% CI: 1.04, 2.98)) and adjusted (OR 1.81 (95% CI: 1.04, 3.16)) models. Answering “yes” to the screening questions regarding reduced dietary intake and weight loss was significantly associated with the incidence of SSI (respectively OR 2.66 (95% CI: 1.59, 4.45) and OR 2.15 (95% CI: 1.23, 3.76)). In conclusion, we demonstrate SSI to occur more often among patients at nutritional risk as compared to those who are not at nutritional risk. Future studies should investigate interventions to prevent both SSI and nutritional risk among surgical patients.

## Introduction

A surgical site infection (SSI) is defined as an infection that has occurred within 30 days after a surgical procedure in the part of the body where the surgery took place, and is one of the most commonly reported health care-associated infections in both European countries and in the U.S. [1, 2]. Such infections are associated with reduced health-related quality of life [3], higher morbidity and mortality [4], and leads to extreme costs for the health care system [3, 5]. SSI is most often a result of contamination during surgery. However, several patient characteristic affect the risk of developing a SSI, including undernutrition, as described in both WHO Guidelines for Safe Surgery and Centers for Disease Control and Prevention’s Guideline for the Prevention of Surgical Site Infection [6, 7].

Although the diagnosis of undernutrition has no commonly accepted definition, the term usually includes conditions associated with low food intake, weight loss and/or low body mass index (BMI) [8]. In the hospital setting, one of the most commonly used screening tools to diagnose patients to be at risk of undernutrition or to already be undernourished is the Nutritional Risk Screening (NRS-2002) [9–11]. In addition to identifying patients to be at nutritional risk, NRS-2002 is able to predict higher treatment costs and one-year mortality in hospitalized patients [12]. Interestingly, only a few and rather small studies (conducted in patient groups having surgery for colorectal cancer (n = 352) [13], major laparoscopic abdominal surgery (n = 75) [14] and pancreaticoduodenectomy (n = 64) (*the latter study only significant results in the unadjusted analysis*) [15]) have demonstrated nutritional risk to be a risk factor for SSI. As NRS-2002 is a well-known, non-invasive and fast screening tool to use in clinical practice, it would be of major interest if it also could identify those with an increased risk of SSI. Thus, we aimed to investigate the association between nutritional risk, as defined by NRS-2002, and the incidence of SSI within 30 days after surgery in a larger, mixed surgical patient-sample.

## Materials and methods

### Study sample

The present study included 1194 surgical patients from Haukeland University Hospital, which is a combined emergency and referral teaching hospital with 1100 beds in Hordaland County in the western part of Norway. In Norway, monitoring the incidence of SSI after five surgical procedures (aortocoronary bypass, cesarean, inserting prosthesis in hip joint (total and hemi prosthesis), colon surgery and cholecystectomy (open and laparoscopic)) through the NOIS-registry regulation (NOIS; Norwegian Surveillance System for Health Care Associated Infections in Hospitals) has been mandatory since 2005 [16]. The registration is coordinated by the Norwegian Institute of Public Health and registered in the NOIS-POSI (POSI; postoperative site infection) database as previously described [17]. In addition to these nationally mandatory registrations, Haukeland University Hospital has monitored SSI for several more procedures since 2004. This is registered as a local quality improvement project, and is further referred to as the local NOIS-POSI database.

Another quality improvement project conducted at Haukeland University Hospital is the regular prevalence surveys of nutritional risk among hospitalized patients. These point-prevalence registrations have been mandatory for the somatic departments and have annually been repeated three to four times since 2008 among non-terminal, non-pregnant and non-bariatric surgical patients ≥ 18 years [18]. Since then, almost 2000 patients have been evaluated and registered in this Nutritional risk database each year.

**Fig 1:**
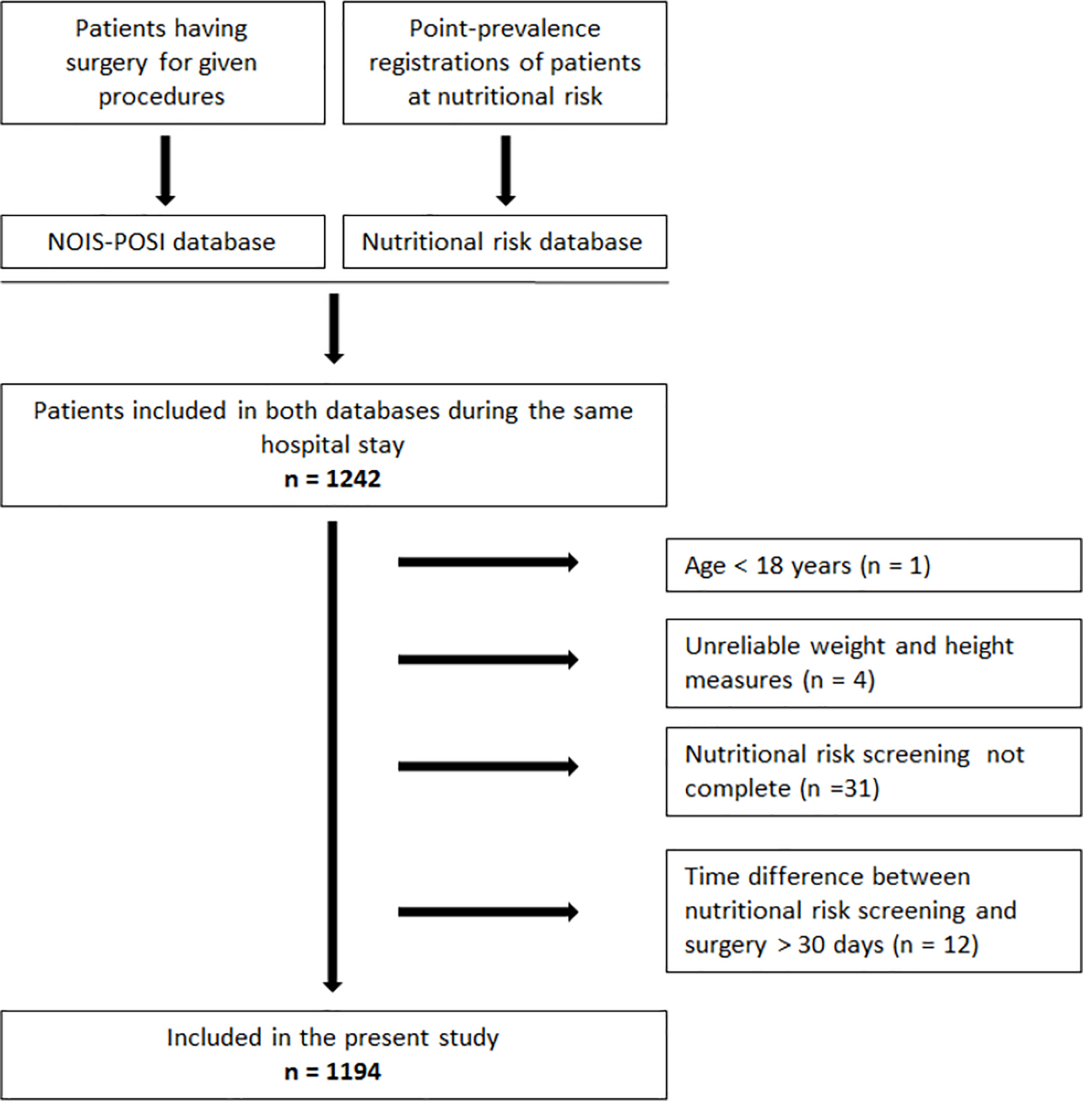
Flow chart of the study sample.

The study population includes patients who during the same hospital stay at Haukeland University Hospital, in the period from 2008 and out 2016, were both registered in the local NOIS-POSI database and the Nutritional risk database. The patients were excluded if the nutritional risk screening was conducted more than 30 days before or after surgery. Patients with an unreliably BMI-value and patients who were not completely screened to achieve the diagnosis “at nutritional risk” were also excluded (Fig 1).

### Ethics

The current study is based on two quality improvement projects that aimed to monitor the incidence of SSI and prevalence of nutritional risk, as well as monitor and improve clinical practice. Such improvement projects do not need to pass Regional Committee for Medical and Health Research Ethics or obtain patient consent according to the Health Research Act, Norway. This current study was approved by the Regional Committee for Medical and Health Research Ethics (2015/2034) before merging data from these two quality improvement projects, and the datasets were anonymized prior to access and analysis. The study is in accordance with the Declaration of Helsinki.

### Assessment of clinical data

The incidence of SSI was registered within 30 days after surgery, either at hospital discharge or at voluntary follow-up mail sent to the patients 25–30 days after surgery. The diagnosis of deep surgical infection and organ/space infection was set by a physician after standardized criteria from CDC/ECDC [19, 20], and superficial SSI was either set by a physician or was self-reported by a patient questionnaire. Non-responders were sent reminders and received telephone follow-up as previously described in detail [21].

A nurse or a nurse assistant used the screening tool NRS-2002 to determine whether the patients were at nutritional risk or not. NRS-2002 is based on four introductory questions on low BMI (<20.5 kg/m^2^), recent weight loss, recently reduced food intake and critical illness [9]. If one or more of these four questions are answered with “yes”, the patient enters the final screening. The final screening gives a total score from 0 to 7 based on more in-depth questions regarding the patient’s nutritional status (score 0–3) and the severity of the patient’s disease in light of nutritional requirements (score 0–3), in addition to one score if the patient is older than 70 years. A total score of ≥ 3 in the final screening identifies patients to be at nutritional risk.

Both the incidence of SSI and the prevalence of nutritional risk were registered in a professional data retrieval system developed by Webport (Webport AS, Grimstad, Norway). Information about age, gender, type of surgery and score from the American Society of Anesthesiologists Physical Status Classification System (ASA-score), which was used to evaluate the patients’ physical status [22], was automatically assigned from the hospital’s patient administrative system.

### Statistical analysis

Analyses were conducted for the total study sample, as well as separately among patients at nutritional risk and patients that were not, and among patients who developed SSI and patients that did not. Summary measures for continuous variables are reported as medians (25^th^ to 75^th^ percentile), and categorical variables are reported as counts (percentages). The Kolmogorov-Smirnov test was used to assess normality of continuous variables. Mann-Whitney U and chi-square tests were used to compare sub-groups as appropriate. Crude odds ratios with 95% confidence intervals were calculated by logistic regression models, and the adjusted odds ratio included age (continuous), gender (male/female), type of surgery (planed less or more than 24 hours (acute or elective, respectively)) and ASA-score (score 1–4). The statistical package IBM SPSS Statistics was applied. All P-values were two-tailed and values < 0.05 were considered as statistically significant.

## Results

### Patients’ characteristics

In total, 1194 patients were included in the present study (Fig 1), and their general characteristics are described in Table 1. Overall, 47.4% were men and the median (25^th^, 75^th^ percentile) age and BMI were 68 (59, 77) years and 26.0 (23.4, 29.1) kg/m^2^, respectively. The minority of the patients had acute surgery (17.2%). Most patients were operated in the musculoskeletal system (52.5%), the digestive system (22.4%) or the coronary arteries (18.3%). An overview of the different surgeries according to the classification of the Nordic Medico-Statistical Committee (NOMESCO) Classification of Surgical Procedures (NCSP) [23] are given in Table 2.

**Table 1:** General characteristics and ASA-score for the study sample and according to patients’ nutritional risk status and incidence of surgical site infections^1^.

		Study sample	Nutritional risk			Surgical site infections		
			*Yes*	*No*	*P*^*2*^	*Yes*	*No*	*P*^*2*^
		n = 1194	n = 170	n = 1024		n = 92	n = 1102	
General characteristics								
	Male, *n (percent)*	566 (47.4)	81 (47.6)	485 (47.4)	1.000	44 (47.8)	522 (47.4)	1.000
	Age, *median (25*, *75 percentile)*	68 (59, 77)	74 (62, 82)	66 (58, 76)	< 0.001	65 (55, 77)	68 (59, 77)	0.057
	Acute surgery, *n (percent)*	205 (17.2)	73 (42.9)	132 (12.9)	< 0.001	18 (19.6)	187 (17.0)	0.624
	BMI, *median (25*, *75 percentile)*	26.0 (23.4, 29.1)	20.6 (18.6, 25.3)	26.5 (24.0, 29.4)	< 0.001	25.5 (22.8, 29.2)	26.0 (23.4, 29.1)	0.754
ASA-score								
	1 or 2, *n (percent)*	719 (60.2)	81 (47.6)	638 (62.3)	< 0.001	56 (60.9)	663 (60.2)	1.000
	3 or 4, *n (percent)*	463 (38.8)	88 (51.8)	375 (36.6)	< 0.001	36 (39.1)	427 (38.7)	1.000

^1^ Missing data: ASA-score (n = 12); BMI (n = 6)

^2^ P-values for differences between patients at nutritional risk or not and patients having an incidence of surgical site infection or not were calculated by using Mann-Whitney U test for continuous variables and chi-square tests for categorical variables

**Table 2:** Overview of the organ system operated in the present study (n = 1194).

Organ system operated^1^	n (%)
Adrenal gland (BC)	9 (0.8)
Palate (EH)	1 (0.1)
Coronary arteries (FN)	218 (18.3)
Diaphragm and gastro-esophageal reflux (JB)	2 (0.2)
Appendix (JE)	7 (0.6)
Intestine (JF)	148 (12.4)
Rectum (JG)	71 (5.9)
Biliary tract (JK)	39 (3.3)
Uterus and uterine ligaments (LC)	70 (5.9)
Vagina (LE)	1 (0.1)
Hip joint and thigh (NF)	474 (39.7)
Knee and lower leg (NG)	153 (12.8)
Trunk (QB)	1 (0.1)

^*1*^ The two first letters of procedure code according to the classification of the Nordic Medico-Statistical Committee Classification of Surgical Procedures [23]

### The prevalence of nutritional risk and the incidence of SSI

In this study, 170 (14.2%) patients were identified to be at nutritional risk. As compared to patients who were not at nutritional risk, these patients were older, had more often acute surgery, had a lower BMI and tended to have a higher ASA-score (Table 1). Ninety-two (7.7%) patients had an incidence of SSI, whereas most of them (55.4%) were classified as deep according to standardized criteria [19, 20]. There was essentially no difference in age, type of surgery (acute/elective), BMI or ASA-score among those who had SSI and those who did not (Table 1).

### The association between nutritional risk and SSI

The incidence of SSI was significant higher among patients at nutritional risk (11.8%), as compared to those who were not at nutritional risk (7.0%) (*p* = 0.047). These results were in accordance with the multivariate adjusted analysis, demonstrating patients at nutritional risk to be 1.81 (95% CI: 1.04, 3.16) times more likely to develop SSI as compared to those patients who were not at nutritional risk (Table 3). Furthermore, the initial screening questions about weight loss and reduced dietary intake the last weeks were significantly associated with the incidence of SSI in both crude and adjusted analysis (Table 3). None of the other questions in NRS-2002 demonstrated such associations.

**Table 3:** The incidence of surgical site infections (SSI) according to nutritional risk status and contents of the nutritional risk screening tool (NRS-2002) [9]^1^.

		Study sample^2^ (n = 1194)	Incidence of SSI	
			*Crude odds ratio (95% CI)*^*3*^	*Adjusted odds ratio (95% CI)*^*3*^^,^^*4*^
*Complete NRS-2002*				
Patients at nutritional risk		170 (14.2)	1.76 (1.04, 2.98)	1.81 (1.04, 3.16)
*NRS-2002 initial screening*				
Four initial questions				
	BMI <20.5 kg/m^2^? (yes)	92 (7.7)	0.67 (0.26, 1.69)	0.62 (0.24, 1.60)
	Has the patient lost weight within the last weeks? (yes)	138 (11.6)	2.14 (1.25, 3.67)	2.15 (1.23, 3.76)
	Has the patient had a reduced dietary intake in the last weeks? (yes)	170 (14.2)	2.62 (1.61, 4.26)	2.66 (1.59, 4.45)
	Is the patient severely ill? (yes)	151 (12.6)	1.15 (0.62, 2.11)	1.17 (0.59, 2.32)

^1^ Missing data for data regarding: BMI (n = 6); weight loss (n = 4); dietary intake (n = 1); severely ill (n = 4)

^2^ n (% of the total study sample)

^3^ Estimate of odds ratio by logistics regression models. Patients with a positive answer (yes) on a question were compared with those with a negative answer (no) on the same question. One and one question entered into the regression model.

^4^ Adjusted for age, gender, acute surgery and ASA-score.

## Discussion

### Principal findings

In this large cross-sectional study among mixed surgical patients, we demonstrated a positive association between nutritional risk and the incidence of SSI, independent of age, gender, type of surgery (acute/elective) and ASA-score. Among the questions used to define nutritional risk, answering “yes” to the ones regarding reduced dietary intake and weight loss seemed to be strongest associated with SSI.

### Clinical relevance

Our results may increase the motivation to systematically identify, prevent and treat undernutrition among surgical patients in accordance with established guidelines [7, 24, 25]. Since both nutritional risk and SSI have adverse effects for the patients’ health [3, 4, 12] and the hospital’s economics [3, 5, 12], implementing NRS-2002 and treating patients who are at nutritional risk may benefit both patients and hospitals. Moreover, considering both the risk for undernutrition and SSI, as well as the fact that about 75% of the SSIs are first identified after hospital discharge [26], it is of major importance that the result of NRS-2002 is forwarded to the patient and/or the primary care institutions.

### Possible mechanisms

A number of factors could underlie the positive association between nutritional risk and SSI. Among the questions used in NRS-2002, “yes” to the one regarding reduced dietary intake the last weeks tended to be strongest associated with the incidence of SSI. This is in accordance with previous studies demonstrating patients answering “yes” to a reduced dietary intake to be more likely for mortality the following year and an increased morbidity, as compared to patients answering “no” to the same question [12]. The reduced dietary intake may be caused by several factors, and the current study did not identify whether the dietary intake decreased prior to or after the incidence of SSI. Regardless timing for the weight loss, key aspects of perioperative care from a metabolic and nutritional point of view includes avoiding long periods of preoperative fasting and re-establishing oral feeding as early as possible after surgery [24]. Moreover, it is recommended to focus on nutritional counseling if indicated by the preoperative testing [25]. A pre- and/or postoperatively low dietary intake or starvation may lead to a delayed wound healing since several nutrients are needed for the healing process [27]. However, another possible explanation of the observed association is that a present SSI decreases the patient’s appetite due to pain or illness.

Furthermore, the present study demonstrated weight loss to be of great importance when predicting the incidence of SSI. Weight loss most often occurs due to a reduced dietary intake, but may also be caused by a catabolic state seen during an ongoing or exaggerated stress response after surgery. Such stress is further associated with infection, poor wound healing and impaired immune function [28]. However, previous studies have demonstrated the risk of SSI to increase with both pre- [29] and postoperative [30] weight loss. Of note, NRS-2002 does not divide between wanted or unwanted weight loss. Some patients may be motivated for weight loss prior to surgeries, like elective aortocoronary bypass or inserting prosthesis in hip joint, whereas others may have unwanted weight loss prior to surgery due to reduced general condition or pain, like surgery for acute hip fracture or illness in the digestive system. Interestingly, there was no observed association between BMI less than 20.5 kg/m^2^ and SSI, indicating weight loss to be a higher risk factor to SSI than low body weight itself.

As compared to the national NOIS-POSI report from 2014 [31], the incidence of SSI and the median age tends to be higher in the present study (respectively 7.7 vs 4.5% and 68 vs 60 years). This may be seen in context since increasing age is a risk factor for SSI [32]. Interestingly, the current study did not observe a significant association between age and SSI, possibly due to a generally elderly study sample. The higher median age in the current study as compared to the national report may be explained by that it only includes those who were a part of the Nutritional risk database (18 years and older). Moreover, the observed differences may also be explained by the fact that the national NOIS-POSI reports do not include patient-reported SSI and only reports data for one year at a time and only includes the five surgery procedures that are mandatory to report in Norway.

In addition, the present study has a lower percentage of patients being at nutritional risk compared to what is previously reported from the Nutritional database (14.2 vs 29.0%) [12]. This may be explained by only including those who were a part of the NOIS-POSI database. Moreover, the current study has a high amount of elective orthopedic patients who generally have a low prevalence of nutritional risk [33]. It should also be mentioned that the previous report from the Nutritional database is based on data from 2008–2009, and the prevalence of hospitalized patients at nutritional risk may have decreased some during the later years due to the hospital’s focus on this area.

### Strengths and limitations

Strengths of the current study include the large study sample. The fact that both monitoring the incidence of SSI and the prevalence of nutritional risk are mandatory for the hospital increases the quality of the study. According to this, the NOIS-POSI database reports over 90% complete follow-up after discharge [26]. Other strengths with the study includes that the staff were trained to conduct the monitors and that the ASA-score was used to adjust for physical status when investigating the association between the nutritional risk status and SSI.

There are some limitations in the current study. First of all, the data material is a selection of two different register databases: There is a selection of the original Nutritional risk database since the current study includes only surgical patients, and there is a selection of the original local NOIS-POSI database since NRS-2002 is not validated for patients being less than 18 years, terminal or pregnant. Despite NRS-2002 is a validated screening tool that in a fast way identifies patients to be at nutritional risk or not [9], it does unfortunately not give any detailed information about the patients’ nutritional status. Moreover, the observed association between nutritional risk and SSI could be partially explained by socioeconomic factors or other variables related to both nutritional risk and SSI. Unfortunately, as the current data material is a combined selection of two different surveillance databases with only a few available variables, we did not have information to evaluate potential confounding by other variables than age, gender, type of surgery (acute/elective) and ASA-score. When using point-prevalence data, the probability of including patients with longer length of stay increases (i.e., length bias). Thus, this may have led to a more ill study population, which can be reflected in the higher number of incidence of SSI, as compared to previously reported. Further, the present study design is not able to describe the causality between nutritional risk and SSI, and could not identify whether the patients were at nutritional risk prior to or after the surgery.

## Conclusions

In conclusion, we demonstrate SSI to occur more often among patients being at nutritional risk as compared to those who are not at nutritional risk. Future studies should investigate interventions to prevent both SSI and nutritional risk among surgical patients.

## Supporting information

S1 File Anonymized data set. The data set is anonymized and has grouped the age and type of surgery variables. Replicated results may thus in some degree differ from what is reported in the manuscript.
(XLS)

## Abbreviations

ASA: American Society of Anesthesiologists physical status classification system
BMI: Body mass index
NCSP: NOMESCO Classification of Surgical Procedures
NOIS: Norwegian Surveillance System for Health Care Associated Infections in Hospitals
NOMESCO: Nordic Medico-Statistical Committee
NRS-2002: Nutritional risk screening tool (2002)
POSI: Postoperative site infection
SSI: Surgical site infection
